# Anthropometric Measurements Usage in Medical Sciences

**DOI:** 10.1155/2015/404261

**Published:** 2015-08-27

**Authors:** Nevin Utkualp, Ilker Ercan

**Affiliations:** ^1^Uludağ University School of Health, Bursa, Turkey; ^2^Department of Biostatistics, Uludağ University Faculty of Medicine, Bursa, Turkey

## Abstract

Morphometry is introduced as quantitative approach to seek information concerning variations and changes in the forms of organisms that described the relationship between the human body and disease. Scientists of all civilization, who existed until today, examined the human body using anthropometric methods. For these reasons, anthropometric data are used in many contexts to screen for or monitor disease. Anthropometry, a branch of morphometry, is the study of the size and shape of the components of biological forms and their variations in populations. Morphometrics can also be defined as the quantitative analysis of biological forms. The field has developed rapidly over the last two decades to the extent that we now distinguish between traditional morphometrics and the more recent geometric morphometrics. Advances in imaging technology have resulted in the protection of a greater amount of morphological information and have permitted the analysis of this information. The oldest and most commonly used of these methods is radiography. With developments in this area, CT and MRI have also been started to be used in screening of the internal organs. Morphometric measurements that are used in medicine, are widely used in the diagnosis and the follow-up and the treatment of the disease, today. In addition, in cosmetology use of these new measurements is increasing every day.

## 1. Introduction

Since ancient times, the human body has been measured for several reasons. During the ancient era, human body measurement was mostly practiced for the figurative arts. Eventually, the practice was adopted by the naturalist field and then by anthropologists to identify human basic morphological characteristics. The term anthropometria dates back to the 17th century in the naturalist field, when it first appeared in the short manual* Anthropometria* by Johann Sigismund Elsholtz [1–3]. The manual seems to be the earliest recorded material that investigated the human body for scientific and medical purposes. It introduced a quantitative approach to seek information concerning variations and changes in the forms of organisms that described the relationship between the human body and disease [4]. Elsholtz proposed that the use of anthropometry constituted a valuable measurement strategy for different fields such as medical practices, physiognomy, the arts, and ethics [3, 5]. In the second half of the century, a strong need for counting and measuring the human body arose, and the representation of the instruments used in clinical practices became vital for the medical field. The* pulsilogium*, which was invented by Sanctorius at the University of Padua, was one of the first instruments in the field and was used to evaluate the pulse rate. During the 18th century, the well-known French anatomist Jean-Joseph Sue, Swiss physiognomist Johann Kaspar Lavater, and German naturalist Johann Friedrich Blumenbach presented valuable research on different issues concerning measurement [6]. At the prompting of these academics, “the season of measurers” began, and practitioners started to believe in the practical application of numbers. Making use of mathematics, geometry, and statistics, anthropologists presented human investigation methodologies and became “anthropometers” [1, 2]. The anthropologists' prior object of investigation was “the skull,” which they believed represented the most important part of the body. The anthropometrical method became more popular in several fields due to the research of Adolphe Quetelet in the 19th century [2]. During this period, the new conceptualization of human diversity advanced this practice for the creation and validation of racial typologies [1].

In the West, the use of measurements and the description of the human body emerged among the artists of classical civilizations; however, more systematic body measurements and records gained importance due to the demands of early modern military organizations [2]. The measurement of the height of individuals, especially young men, became the basic procedure used to classify them as appropriate or not for military recruitment. Through the end of the 19th century, anthropometry became a new tool for clinical practices and taxonomy as public health measurements gained importance. In the 19th and 20th centuries, anthropometry manifested in the measurements of weight, circumference, stature, and skinfold thickness that were used to identify environmental influences that impacted child growth [4].

Because ancient anthropometric research was a relatively current concept, the related medical literature concerning nutrition and physical growth served as a valuable theoretical source. Hence, the biomedical literature of the World Health Organization (WHO) was regarded as one of the best sources that represented general health conditions within a society [3].

Because of its use as a measurement of physiological and developmental human growth, anthropometria appeared in several clinical practices that utilized instruments such as the manometer, sphygmograph, hemocytometer, hemoglobinometer, and spirometer [2]. The need for these measurements stemmed from the interaction between several intricately linked concepts, including nutrition and infection, psychosocial stress, food contaminants, hypoxia, and pollution [1]. Factors mostly linked to socioeconomic status and poverty indicated that body size was a signal for the quality of life. Thus, anthropometric practices could be used as a tool for social welfare, whereas factors such as culture, society, behavior, and the political economy played important but distal roles in the outcomes of growth and body size [1, 3, 5].

## 2. Historical Development of Anthropometry

Over the ages, all civilizations have been interested in the human body. Artists in particular have reflected the effects of this interest in their works.

In the ancient Egyptian, Greek, and Roman civilizations, famous artists used male figures in their artwork (i.e., pictures and statues) with the desire to represent issues such as beauty, virtue, independence, military power, and authority [6, 7].

In the ancient era, artists were interested in the depiction of body parts based on reciprocal proportions. Artists believed that the human body represented as “an ideal human figure” had specific proportions between its constituent parts. Throughout history, these proportions were considered to be canon. In practical use, any given part of the human body could be chosen for measurement and proportioned to the other parts due to the absence of standardized measurement units such as the meter, centimeter, or millimeter. Therefore, any given human body part could be described as a “unit of measurement” (module). These measurement units contained various modules such as the length of the feet, length of the hand, and height of the head [5, 8, 9].

Throughout history there have been studies related to the “human body” branches of art (i.e., sculpture and painting) as well as studies related to anatomy in the field of medicine. In the three most well-known ancient civilizations, scholars evaluated the “human body” using the concepts of canon and modules [6, 10].

## 3. Anthropometric Measurements in Ancient Civilizations

### 3.1. Egyptian Civilization

The first known dissections with the aim of learning (III century BC) were performed by scholars in Egypt [7]. In the most ancient cannon, “length of feet” (LF) was used as the module. Human figures drawn on the walls of the pyramids by Egyptian artists were depicted with heights six times longer than the length of their feet; however, when the artists noticed that the proportions did not reflect reality, they adjusted the height of taller human figures to a height equivalent to seven feet. According to our present arithmetic knowledge, they proportioned the horizontal lines based on height and the vertical lines based on the width of the human body [7, 9].

### 3.2. Ancient Greek Civilization

The most famous artist of this era was Polykleitos. Polykleitos evaluated the human body and wrote the first known artistic anatomy book. The renowned scholars used the “width of hand” (WH) as a module and described the proportions he used between various body parts and the width of hand as well as the inequalities. During the period of Greek civilization, for the first time multiple equalities were used in drawings of the human body between the longitudinal, oblique, and transversal dimensions [7].

### 3.3. Roman Civilization

Roman artists and scholars further developed studies of the “human body.” Moreover, some equalities were described after a human figure in the college position was placed in a square frame. Because notables of the era such as Leonardo da Vinci found that the human figure in the college position had an equal length and width, human paintings were often performed using a square frame [7, 9, 10]. Artists during the era of the Roman Empire continued these studies by merging art with anatomy and quietly exploiting mathematics [11].

### 3.4. Anthropometric Measurements during the Renaissance

Great artists of the renaissance (Leonardo da Vinci and Albrecht Dürer) created many works based on these rules and proportions. Works related to the human body were developed according to rules that were considered to represent classical anthropometrical measurement techniques [7].

(i) The renowned renaissance artist Leonardo da Vinci was interested in both art and sciences. He performed cadaver dissections and notated his measurements, notes, and drawings with the attention to detail of a scientific investigator. For the first time in history, he investigated the human face, head, neck, and other related parts in detail, mainly following the “Polykleitan theory.” He worked on a drawing belonging to Vitruvius, and after rigorous investigation of this work he demonstrated his success in this field. Indeed, the “Vitruvian man” became one of his most renowned works [7, 9].

(ii) Durer was a versatile artist and architect who worked in both the mathematics and anatomy fields. He was born in Germany and examined both the male and female figures from the perspective of science and art. However, in his era dissection was not allowed in Germany, so his work relied on the use of live models and examinations of the literature. He also has investigated the positions of the internal organs and depicted the projections of the spleen in his work. His most famous work titled “Adam and Eve” showed his incredibly rigorous calculations [7].

### 3.5. Anthropometric Works in the “20th” Century

After the 19th century, the concept of the “average” male figure was developed based on comprehensive measurements. In the early 20th century, the French doctor of medicine and painter-sculptor Paul Richer performed one of the most detailed and scientific studies of the postrenaissance era due to his use of anthropometric methods. He described the “average human figure” based on comprehensive measurements rather than the “ideal human figure.” He chose “height of head” as the module and depicted the front and the back view. Additionally, he explained human anatomy in the context of the medial and lateral views of the extremities [1, 5, 10].

Morphometrics, a branch of anthropometry, is the study of the size and shape of the components of biological forms and their variations in populations [11]. Morphometrics is a field concerned with studying variations and changes in forms (i.e., size and shape) of organisms; morphometrics can also be defined as the quantitative analysis of biological forms. The field has developed rapidly over the last two decades to the extent that we now distinguish between traditional morphometrics and the more recent geometric morphometrics [4].

#### 3.5.1. Traditional Morphometrics

In traditional morphometrics, it is not possible to recover the shape of the original form using the usual data matrices of distance measurements, even as an abstract representation. The overall form is neither archived nor used in the analysis. For example, a researcher may know that several measurements share a common landmark, but this information is not used in the multivariate analyses. As a result, the analyses cannot be expected to be as powerful as they could be if that information were taken into account [4, 11].

Traditional morphometrics consisted of applying multivariate statistical analyses to sets of traditional measurements between points with biological and anatomical meanings to define shapes called landmarks. These measurements usually represented the lengths and widths of structures and the distances between certain landmarks, which are described as the points of correspondence on each matching object between and within populations. Sometimes angles and ratios were used [11, 12].

When multivariate morphometrics was combined with both quantitative morphology and multivariate statistics, several difficulties still remained. As an example, many ways of size correction were proposed, but there were great debates about which method should be utilized [4, 11]. It was important due to little different results caused by different size correction methods. Second, homology of linear distances was difficult to be evaluated due to insufficiency of homologous points about defining many distances (maximum width, etc.). Thirdly, similar set of distance measures may be obtained from two different shapes because data did not include location of each distance measurement which were relative to the other distance measurements. Traditional morphometrics does not allow recovering shape of original form from usual data matrices even if it is an abstract representation. Archives and analyses did not include whole form. A researcher may know the common landmark shared by several measurements; however, this knowledge has no role in multivariate analyses. As a result, analyses will not be powerful as the condition which information were used in [4, 11–13].

#### 3.5.2. Geometric (Modern) Morphometrics

In the 1960s and 1970s, biometricians began applying multivariate statistical analyses to sets of traditional measurements. Geometric morphometric methods are more valid than traditional morphometric methods in protecting morphological information and permitting the analysis of this information. For morphometrics to fulfill its promise of fusing geometry with biology there must be equal emphasis on the two components. Morphometric techniques need to be designed and applied with biology in mind, and the quantitative results must be directly interpretable using biological methods [11, 13].

In geometric morphometrics, biological shape is defined via transformation of the original shape, which is selected as a reference shape. Thompson proposed the idea in 1942, and although the method was attractive and promising for the analysis of biological shapes, the method did not have an analytical procedure. With the advent of computers, applications for morphometric analysis based on Thompson's idea became possible. Data are recorded to represent the geometry of the structure being studied [11]. These data are in the form of two-dimensional (2D) or three-dimensional (3D) coordinates of morphological landmark-points. The estimates of the parameter of the fitted function can then be used as variables in standard univariate and multivariate statistical analyses [12]. The coordinates are much more useful than traditional measurements, and the usual distance measurements can be computed from the coordinates [11, 12, 14]. Using landmark coordinates, concise encoding of all information in any subset of distances or angles between them is possible. Analysis and visualization which is on coordinate-based approaches are called complete retention of geometric information from data collection. Within geometric morphometrics, collecting information concerning the location of different points as landmarks addresses comparisons between organic forms. Considering points as homogenously distributed on the organism and have some biological meaning, a set of homologous points, landmarks provide information of biological life forms [11–13].

The fundamental advantages of geometric morphometrics over traditional approaches (i.e., multivariate morphometric techniques) include the development of powerful statistical methods based on models that are used to examine the shape variation of all configurations that correspond to morphologic landmark locations. Indeed, in many biological or biomedical studies, the most efficient way to analyze the forms of whole biological organs or organisms is by registering landmarks [4]. Many studies in medicine are related to the examination of the geometrical properties of an organ or organism. In these studies, statistical analysis consists of the quantitative or qualitative measurement of given values; for example, recently a given organ or organism's appearance or shape has been used as the input data for the development of imaging techniques [13]. Commonly, quantitative or qualitative data sets used for statistical analysis consist of measurement values. In recent times, following the development of imaging techniques an organ or organism's appearance or shape began to be used as the input data [4]. In these studies, the statistical analysis consists of the quantitative or qualitative measurement of the given values.

For over 50 years, qualitative morphometric techniques have been used within limits to assess bone density. Grading systems for the spine and proximal femur were developed with the aim of characterizing the severity of bone loss. However, because the use of such systems could cause highly subjective interpretations, the inclusion of a series of reference radiographs is recommended. Quantitative morphometric techniques are repeatedly used for imaging of the spine or proximal femur with X-rays. However, some measurement parameters were required for these techniques to produce a quantitative assessment of the severity of bone loss [15–17].

## 4. Radiological Development of Imaging Modalities

Throughout history, many studies have focused on the human body, especially with the aim of identifying anatomical, physiological, and pathological features of the internal organs. Among these studies, those related to imaging modalities of internal organs are especially very valuable [18, 19]. During his work with cathode ray tubes in 1895, German physicist Wilhelm Conrad Röntgen noticed radiating rays when high-voltage electric current passed through a Crookes tube; Röntgen named them unknown rays (X-rays). On December 22, 1895, Röntgen obtained an image of his wife's hand following 15 minutes of irradiation. These rays were identified as very high frequency electromagnetic waves with light bursts as florescence. X-rays can pass through soft tissues and partially penetrate into dense tissues such as bone. This process enabled internal views to be obtained as images from living organisms. Röntgen presented his invention to the Physical Medicine Society in Germany, and two weeks later he obtained images of his own upper and lower teeth using irradiation on black paper and a glass photography plaque wrapped with plastic. These images represented the first radiography images. The first medical X-ray radiography (Röentgen graphy) in history was also obtained during these experiments, and Röntgen officially announced his important discovery on December 28, 1895. Although potential radiation hazards due to the use of X-rays had been ignored, the dentist Frank Harrison reported skin peeling and hair loss in his patients due to the use of X-ray radiography [15, 16].

In Turkey, the usage of X-rays in the field of medicine was first performed by medical students Esat Feyzi and Osman Rifat. Both students detected bullets in wounded soldiers during the Ottoman-Greece battle using radiography [20–24]. One of the first studies concerning X-rays was performed by M. Hubert. In this study, Hubert evaluated the physiological and pathological values of kidneys collected from different species of animals. Rich et al. studied the X-ray sensitivity of human tumor cell. Both Rich et al. and Taoka and Shuloeva provided examples of roentgenological studies of pulmonary function [22, 23, 25].

## 5. Computerized Tomography (CT)

The first quantitative CT measurement was proposed by Johann Radon. In 1972, J. N. Hounsfield scanned a section using thin and weak X-rays and turned the result into an image after computer evaluation by reading the signals in the scintillation chamber. Using this technique, a cross-sectional image could be obtained from anywhere in the body. Investigations of the CT accessibility of tissues and body regions showed that CT is more successful in imaging bone tissue than soft tissues due to its working principles and design. This invention was an important development for the imaging of brain and malignant tumors [26, 27].

Quantitative computed tomography (CT) is used for quantifying bone mineral density (BMD) in the spine, proximal femur, forearm, and tibia as a three-dimensional nonprojectional technique. It has several advantages over other densitometric techniques, including the ability to separate the cortical and trabecular bone, the fact that degenerative changes in the spine cannot affect the volumes of interest (VOI), and the ability to determine 3D geometric parameters [26, 28].

## 6. Magnetic Resonance (MR) Imaging Technique

The identification of spin-based physic resonance by Wolfgang Pauli in 1920 initiated the first attempts to obtain images using the MR technique. Quantitative measurements in this field were first performed by physicists Bloch and Purcell. In their experiments, they demonstrated that atoms with one nucleon in their core were affected by the magnetic field and that the orbit of the atomic cores was changed in response to the magnetic field. For a long time, this finding was applied solely to the field of physics. Then, in 1970 Paul Lauterbur obtained a clear MR image. The first diagnosis using this modality was performed by Hawkes et al. in 1980. Currently, the ability to obtain fast and quality images of internal organs using the MR technique and the relatively low risk of side effects has led to its common use both internationally and nationally [26–30].

## 7. Current Utilization of Three-Dimensional Imaging

Currently, the direct calculation of the measurements of morphometric quantitative area shapes has been made possible by utilizing various programs after the common usage of MRG. Due to its imaging capacity on multiple planes, absence of ionizing radiation, and utilization for the diagnosis of mediastinum, this method has an important place in the field of medicine [29].

Mathematical analyses are used to identify the shape of an anatomic region in the human body. These evaluations are performed using optic measuring methods with 3D imaging modalities. These methods are especially important for quantitating data in the complex anatomical structures of the human body. The assessment of the validity and safety of these data has led to improvements in human health and quality of life [31].

The most commonly used imaging modality trio today includes the PET/CT modalities. In addition to imaging structures in the human body, these modalities can also detect exact tumor locations and biological properties that are essential for diagnoses in cancer patients [28, 30].

Anthropometric measurements are important for the evaluation of morbidities of individuals in society and thus meet the requirements of that society. For human health, the field of medicine requires constant development and renewal. Throughout history, anthropometric measurements were improved as details of human anatomy were discovered, until the field reached today's standards. In recent years, the utilization of many new measurement devices for clinical use and primary studies has inevitably led to improvements in measurement parameters and techniques [6, 7].

In the eras of the Ancient Egyptian, Greek, and Roman civilizations, artists made detailed evaluations of the human body. Artists of the renaissance period created ideal ratios in their works using mathematical methods (i.e., canons and module measurement). The “golden ratio” that was used by Leonardo da Vinci in his drawings currently remains the norm for beauty. In this ratio, anthropometric data and ratios are used to compare the ratios of disproportions present on the face [7].

A tendency towards plastic surgeries has become widespread over the past several years. Interventions related with this field include corrections of congenital malformations as well as various optional modifications on individual's bodies. Anthropometries of the human body and especially the face are used for the identification of these disproportions. Therefore, more standardized and purpose-oriented measurements in the field of plastic surgery are important for a more objective evaluation of human bodies [32].

## 8. Cosmetology

The use of imaging techniques in facial cosmetics is an undesirable feature caused by extrinsic photo damage and the intrinsic aging process [33]. A decrease in wrinkle severity has become a very important evaluation criterion in aesthetic dermatology for the assessment of the success of rejuvenating treatments. Many quantification methods have been developed to analyze wrinkles. The comparative evaluation of modern scales and 3D images can lead to a further understanding of facial wrinkles and may elucidate the connection between clinical assessment and appraisal using biophysical measuring methods. Luebberding et al. investigated facial wrinkles in a study designed to compare clinical ratings and 3D fringe projections [34]. Jiang et al. [35] used the SWIRL (Stephens wrinkle imaging raking light) method as an example. The use of this method represents a step towards better understanding of the actions and changes produced by prescription and cosmetic wrinkle treatment products and medical procedures [35]. Another branch of medicine using imaging techniques is breast cosmetics. However, the concept of breast size itself remains controversial. Breast volume and breast density must be distinguished, and the appropriate measurement, whether subjective reporting, cup size, mammographic assessment, or three-dimensional imaging, remains unclear [26]. Ultrasound and mammography are useful imaging techniques for the assessment of reconstructed breasts in symptomatic settings. Magnetic resonance imaging of the breast is another important diagnostic technique that is useful for breast cancer. Its performance is indicated in several situations, including staging of the disease and treatment planning [27]. MR imaging is the most accurate of the three preoperative imaging modalities in assessing the size and number of malignant lesions in the breast. The studies of Faermann et al. [29] were the first to assess the tumor-to-breast volume ratio measured by MRI and to correlate it to the type of surgery selected for the patient (i.e., breast conservation or mastectomy) [29]. To evaluate the comparative accuracy of magnetic resonance (MR) imaging relative to mammography and ultrasonography (US) for the assessment of the extent of breast tumors, Yımaz et al. reviewed the findings of Boetes et al. [28] and Fischer et al. [30] and suggested that the sensitivity and specificity of US and MRI exams for detecting local recurrence were higher than clinical examinations [7, 28, 30]. Furthermore, MRI plays an important role in treatment planning and is more objective in determining the response of tumoral lesions to systemic treatment. The use of 3D imaging and computerized measurements brings a new dimension into surgical planning. Indeed, studies showed that the portrait 3D platform create in cosmetology [35].

Today, many fields, including plastic surgery, depend on photo documentation as a crucial part of both clinical practice and medical education. The most recent advancement in breast plastic surgery is ideally suited for 3D technology. The portrait 3D breast imaging system provides a highly reproducible 3D tool for measuring breast volume and simulating breast augmentation [33].

## 9. Conclusion

The main reasons for the widespread use of statistical shape analysis in medicine include the fact that geometric morphometric methods are more valid than traditional morphometric methods. Advances in imaging technology have resulted in the protection of a greater amount of morphological information and have permitted the analysis of this information. There is hope that advances in both screening and diagnostic technology will ultimately have a positive impact on treatment. Furthermore, the use of these treatment modalities for cosmetic use has been rising.
